# Predictors of survival after hepatic resection among patients with colorectal liver metastasis

**DOI:** 10.1038/sj.bjc.6604093

**Published:** 2007-12-11

**Authors:** X Wang, D L Hershman, J A Abrams, D Feingold, V R Grann, J S Jacobson, A I Neugut

**Affiliations:** 1 Department of Biomedical Informatics, College of Physicians and Surgeons, New York Presbyterian Hospital, Columbia University, New York, NY 10032, USA; 2 Department of Medicine, College of Physicians and Surgeons, New York Presbyterian Hospital, Columbia University, New York, NY 10032, USA; 3 Department of Surgery, College of Physicians and Surgeons, New York Presbyterian Hospital, Columbia University, New York, NY 10032, USA; 4 Department of Epidemiology, Mailman School of Public Health, Columbia University, New York, NY 10032, USA; 5 Herbert Irving Comprehensive Cancer Center, College of Physicians and Surgeons, Columbia University, New York, NY 10032, USA

**Keywords:** colorectal cancer, hepatic resection, elderly, SEER-Medicare, metastases

## Abstract

Studies suggest improved survival following resection of colorectal cancer liver metastases (CLMs). We investigated predictors of survival among patients with CLM who underwent hepatic resection using the SEER-Medicare database to identify patients ⩾65 years diagnosed with CLM, 1991–2003, who underwent hepatectomy. Cox proportional hazards models were used to identify factors associated with survival after hepatectomy. Of 923 patients with CLM who underwent hepatectomy, 514 were stages I–III and developed CLM>6 months after diagnosis (metachronous), and 409 were stage IV with CLM at diagnosis (synchronous). From the date of hepatectomy, 5 year survival was 22%; younger age, being married, female gender, surgery in an NCI-designated cancer centre, fewer comorbidities, fewer positive lymph nodes, and lower grade were associated with improved survival. Both 5-fluorouracil (5FU)-based chemotherapy and hepatic arterial infusion (HAI) of floxuridine-based chemotherapy following hepatectomy improved survival (HR=0.62, 95% CI: 0.50–0.78; HR=0.51, 95% CI: 0.28–0.97, respectively) in the synchronous, but not metachronous, group. The HR for overall mortality was higher in hospitals with a high *vs* low procedure volume (0.75, 95% CI: 0.58–0.94). A substantial subgroup of patients with CLM who undergo hepatectomy experiences long-term survival. High hospital procedure volume and use of 5FU-based or HAI-based chemotherapy after resection were associated with improved prognosis.

Colorectal cancer is one of the most common cancers worldwide and remains the second most common cause of cancer death in the United States (Parkin *et al*, 2005). The liver is the most common distant site of metastasis from colorectal cancer (70%) and is often the only organ affected (Scheele *et al*, 1990; Stangl *et al*, 1994). Recent reports indicate that liver metastases occur in more than 50 000 patients with primary colorectal cancer each year in the United States (Foster, 1978; Stangl *et al*, 1994; Blumgart and Fong, 1995).

Patients with liver metastases from colorectal cancer (CLM) have a poor prognosis. Following diagnosis, the median survival of untreated patients with CLM is 6–12 months. The median survival is more than 24 months in patients treated with chemotherapy, but they remain unlikely to be cured (Cady *et al*, 1970; Wilson and Adson, 1976). Surgical resection of liver metastases is currently considered first-line treatment, with alternative therapies, such as radiofrequency ablation, reserved for those lesions that are not amenable to resection (Gillams and Lees, 2005). In recent years, however, advances in early diagnosis and surgical procedures appear to offer better long-term survival and cure in patients with limited CLM who are resectable, with a reported median survival of 35–69 months. Large series report 5-year and 10-year survival rates as high as 51 and 27%, respectively, following hepatectomy in this setting (Scheele *et al*, 1995; Fong *et al*, 1999; Bolton and Fuhrman, 2000; Sugawara *et al*, 2001; Choti *et al*, 2002; de Santibanes *et al*, 2002; Adam *et al*, 2003).

The factors, which predict prognosis and survival after hepatic resection for CLMs, have not been well defined. Furthermore, while systemic chemotherapy is frequently administered after resection, the optimal regimen and its contributions to survival outcomes are inconclusive (Kemeny *et al*, 2002; Portier *et al*, 2006; Yoshida *et al*, 2006). We, therefore, conducted a population-based study to investigate the impact of demographic, clinical, and treatment characteristics on survival after hepatic resection for CLMs.

## PATIENTS AND METHODS

### Data sources

The primary data source for this study is a database created through cooperative efforts by the National Cancer Institute's Surveillance, Epidemiology, and End Results (SEER) cancer registry; the Center for Medicare & Medicaid Services (CMS); and the National Cancer Institute. Developed in 1993 (Potosky *et al*, 1993), the SEER-Medicare database links information on patient demographics, tumour incidence, histology, location, stage, treatment, and survival from the SEER programme, a cancer registry comprising a representative sample (approximately 25% at the time of this study) of the US population, to the individual medical insurance claims for cancer-related services (office visits, diagnostic testing, and treatments) provided to patients 65 years of age and older collected in the CMS Medicare database. The SEER-Medicare database has been described in detail (Klabunde *et al*, 2000) and has been validated previously for documenting the use of surgery (Cooper *et al*, 2002) and chemotherapy (Warren *et al*, 2002). This study was approved by the Columbia University Institutional Review Board.

### Patient selection criteria

We identified all persons within the SEER-Medicare database who had a diagnosis of colorectal cancer from 1 January 1 1991 to 31 December 31 2003, 65 years of age or older, who at diagnosis or later developed a diagnosis of liver metastasis (International Classification of Diseases (ICD) 9th code: 197.7), and who underwent a hepatic resection. Patients who were enrolled in a health maintenance organisation at any point of time from 12 months before to 8 months after the diagnosis of CLM were excluded due to the incompleteness of this information in the database. We also excluded individuals with any gaps in Medicare Parts A and B coverage during the study period. Subjects whose metastases were coded within 180 days of their primary diagnosis with American Joint Committee on Cancer (AJCC) stages I–III were excluded from the study, since the primary diagnosis may have been mis-staged (*N*=85). Patients were further classified as synchronous (*n*=409) if a patient was diagnosed with AJCC stage IV or colorectal cancer with liver metastasis, and metachronous (*n*=514) if the patient was diagnosed with stages I–III colorectal cancer and subsequently developed liver metastases after 180 days.

### Measurement of study variables

#### Patient characteristics

Subjects were categorised by age group at diagnosis, year of diagnosis, race/ethnicity, gender, marital status, number of positive lymph nodes of primary colorectal cancer, tumour grade (well/moderately differentiated or poorly differentiated) of primary cancer, comorbidity score, residence (metropolitan or non-metropolitan area), the type of hospital in which they received care (teaching or non-teaching, NCI-designated cancer centre) and the socioeconomic status (SES) of their census tract/zip code. We obtained data on age, race, gender, marital status, surgery, stage, tumour grade, lymph nodes, type of hospital, and area of residence from the SEER database, NCI Hospital file, and AMA files, and data on comorbid conditions and treatment from Medicare.

We generated an aggregate SES score from a hierarchy of income data from the 2000 census. Patients were ranked on a 1–5 scale, with 1 as the lowest value, based on a formula incorporating as many of the following as were available: median income in the census tract of residence, median income in the zip code of residence, census tract per capita income, zip code per capita income; patients for whom all of these values were missing were assigned to the lowest SES category.

To assess the prevalence of comorbid disease in our cohort, we used the Klabunde adaptation of the Charlson comorbidity index (Charlson *et al*, 1986; Deyo *et al*, 1992; Klabunde *et al*, 2000). Medicare in-patient and outpatient claims were searched for ICD-9-CM diagnostic codes, indicating a history of myocardial infarction, congestive heart failure, peripheral vascular disease, cerebrovascular disease, dementia; chronic pulmonary disease, connective tissue disease, mild-to-severe liver disease, diabetes with/without end-organ damage, haemiplegia, moderate-to-severe renal disease, or AIDS, in the Medicare files from 12 months before to 1 month after their diagnosis of cancer. Each condition was weighted, and patients were assigned a score based on the Klabunde–Charlson index (Klabunde *et al*, 2000, 2002).

#### Treatment characteristics

Information on hepatectomy was obtained by using an algorithm that searches and combines the ICD-9-CM codes (50.21, 50.22, and 50.29 (wedge resection), 50.3 (lobectomy of liver), and 50.4 (total hepatectomy)) and Current Procedural Terminology (CPT) codes (47 120 (resection of liver and partial lobectomy), 47 122 (trisegmentectomy), 47 125 (total left lobectomy), and 47 130 (total right lobectomy)). The hospital procedure volume was defined based on the number of operations performed by the given hospital in Medicare databases during the study period as previously validated (Begg *et al*, 1998). The hospitals were ranked by procedure volume: low (1–8), intermediate (9–29), and high (30–125).

We defined other treatment exposures according to previously published criteria for the SEER-Medicare database. Chemotherapy exposure was derived through Medicare records and ICD-9 codes (diagnosis and procedural), CPT and Healthcare Common Procedure Coding System, and revenue centre codes. The status of chemotherapy was classified as follows: hepatic arterial infusion (HAI) of floxuridine (FUdR) (J9200 and C9426) with 5-fluorouracil (5FU)-based (J9190) systemic chemotherapy, 5FU-based (J9190) systemic chemotherapy alone, or no chemotherapy.

### Survival

Survival time was analysed as the number of months from the hepatectomy date to the Medicare date of death. Those alive at the end of the follow-up period were classified as censored and contributed the time interval from their diagnosis date to the end of follow-up to the survival analysis. Overall survival is tracked on a yearly basis for each subject in the SEER database.

### Statistical analysis

Statistical analyses were conducted using SAS version 9.1. We developed Cox proportional hazards regression models with all-cause mortality as the dependent variable and type of chemotherapy, age at diagnosis, race, SES, marital status, year of diagnosis, stage of disease, tumour grade, type of hospital, and comorbidity score as the independent variables for all subjects who had hepatectomy. Type of liver resection was not included in the model, as it is not an independent predictor of disease recurrence or overall survival after surgical resection of metastases (Hamady *et al*, 2006; Zorzi *et al*, 2006). The Kaplan–Meier analysis was used to plot survival curves.

## RESULTS

### Baseline descriptive statistics

We identified 514 metachronous CLM patients and 409 synchronous CLM patients, aged 65 years or older, diagnosed between 1991 and 2003 (Table 1). Most of the patients in the sample were non-Hispanic whites (87%), and most resided in metropolitan areas (91%). The overwhelming majority of patients (91%) received care in teaching hospitals.

Some differences were noted in demographic, clinical, and treatment characteristics between the two groups. More metachronous than synchronous patients received their care in teaching hospitals (93 *vs* 89%, *P*=0.03) or NCI-designated cancer centres (36 *vs* 31%, *P*<0.001). Similarly, more metachronous patients received their resections in hospitals with high procedure volumes than synchronous patients (47 *vs* 31%, respectively; *P*=0.002).

After hepatectomy, fewer metachronous patients received adjuvant chemotherapy than synchronous patients (36 *vs* 62%, *P*<0.001). The use of hepatic infusion therapy was similar in both groups. The median follow-up after resection for metachronous patients was 26 months (range: 1–143 months), and 339 died during the follow-up period. For synchronous patients, the median follow-up was 25 months, and 296 died during the follow-up.

### Mortality and survival

In a multivariate Cox proportional hazards model, younger age, fewer positive lymph nodes, and the receipt of care at hospitals with higher procedure volume were associated with a reduction in overall mortality in the overall sample (Table 2). Female gender, receipt of care at an NCI-designated cancer centre, and receipt of adjuvant chemotherapy for the primary tumour were associated with improved survival in metachronous CLM patients. Among synchronous CLM patients, single marital status, higher comorbidity score, greater number of positive lymph nodes in the primary colorectal cancer specimen, and poorly differentiated tumours were all associated with increased mortality. Systemic 5FU-based chemotherapy decreased mortality (HR 0.62, 95% CI: 0.50–0.78) in the synchronous metastasis group. Similarly, HAI with FUdR improved survival in the synchronous CLM group (HR 0.51, 95% CI: 0.28–0.90), but not among the metachronous patients.

**Figure 1:**
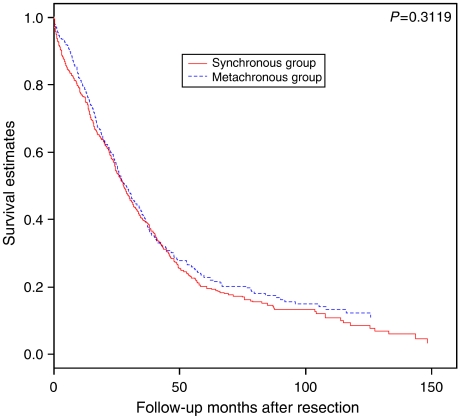
Kaplan–Meier curves of overall survival in metachronous and synchronous colorectal cancer liver metastases after liver resection.

In an unadjusted Kaplan–Meier analysis, the 5-year overall survival from hepatectomy was 25% among metachronous CLM patients and 19% among synchronous CLM patients (*P*=0.3119 for log-rank test) (Figure 1).

## DISCUSSION

In this population-based study, we found that hepatic resection for colorectal liver metastases resulted in an overall 5-year survival of 22% in elderly patients. Those who presented with liver metastases at the time of diagnosis had similar survival to those subjects who had a recurrence in the liver after an initial diagnosis of stages I–III colorectal cancer. In addition, after adjustment for known confounders of mortality, 5FU-based systemic chemotherapy alone, HAI with FUdR with systemic chemotherapy, and higher hospital procedure volume were each associated with improved overall survival.

A recent population-based study investigated the benefits of hepatic resection in colon cancer patients diagnosed with liver metastases (Cummings *et al*, 2007). In this study, the investigators evaluated the outcomes of patients who underwent resection to those who did not. They found that stage IV colon cancer patients who underwent hepatic resection of CLMs had improved survival compared to those who did not (HR 2.78, 95% CI: 2.53–3.04). The conclusions to be drawn from this study were limited due to the inability to account for various factors that influence the decision to perform hepatic resection, such as the presence of metastases at other sites, the number of liver metastases, and the sizes of the liver metastases. They also found that patients who presented initially with stages I–III disease (metachronous) had better survival than those who had hepatic metastases at the time of diagnosis (synchronous), in contrast to our findings. However, they measured survival from the time of the original cancer diagnosis, and not from the time of hepatic resection. Thus, the lead time of the progression from stages I–III till the occurrence of the liver metastases and surgery would bias the results and increase the observed survival of the metachronous group.

Presently, there is no standard guideline for CLM patients after liver resection. There are a few randomised clinical trials published that evaluate the survival effects of HAI of FUdR and/or intravenous systemic chemotherapy after hepatic resection (Lygidakis *et al*, 1995; Asahara *et al*, 1998; Lorenz *et al*, 1998; Lorenz and Muller, 2000; Tono *et al*, 2000; Kemeny *et al*, 2002; Kemeny and Gonen, 2005; Portier *et al*, 2006). Most of these studies, however, have limited statistical power due to small sample sizes (less than 100 subjects) or slow accrual. Among five studies in which HAI with FUdR chemotherapy was compared with resection alone, disease-free survival was reported to be better in patients with adjuvant HAI of FUdR after resection in three studies (Lygidakis *et al*, 1995; Asahara *et al*, 1998; Kemeny *et al*, 2002). An overall survival benefit was observed in two of these studies (Lygidakis *et al*, 1995; Asahara *et al*, 1998). The trial by Portier *et al* (2006) evaluated the survival benefits of systemic intravenous adjuvant chemotherapy and resection alone. Intravenous systemic chemotherapy following hepatectomy improved disease-free survival significantly (HR 0.66, 95% CI: 0.46–0.96). A trend to improved overall survival was also observed, but was not statistically significant (HR 0.73, 95% CI: 0.48–1.10). A very recent study presented in abstract form from the EORTC randomised 364 patients to either surgery alone or perioperative FOLFOX (six cycles preoperatively and six cycles postoperatively) (Nordlinger *et al*, 2007). There was an improvement in progression-free survival at 3 years for the chemotherapy arm, but not in overall survival. Our observation that both HAI with FUdR and systemic chemotherapy improved overall survival in the synchronous metastasis group was consistent with these trials. The large sample size in our study could be the reason that we observed statistically significant survival benefits.

Interestingly, in our analysis, neither 5FU-based chemotherapy nor HAI with FUdR use resulted in a survival benefit in patients with metachronous CLM. No prior study has evaluated the differential benefits of chemotherapy in synchronous *vs* metachronous metastases separately. Our data suggest that 65% of metachronous CLM patients had adjuvant systemic chemotherapy administered following the initial resection of their primary tumours. One possible explanation for the lack of a survival benefit for post-hepatectomy chemotherapy could be due to chemoresistance. Drug resistance has been reported to cause treatment failure in 90% of metastatic cancer patients (Longley and Johnston, 2005). Although not directly studied, their prior exposure to 5FU-based chemotherapy may influence the efficacy of chemotherapy at the time of liver recurrence. In contrast, the synchronous patients are chemonaive and being treated for the first time and so they may be more sensitive to chemotherapy.

A recent investigation found that in-hospital mortality after hepatic resection was higher at lower volume hospitals. However, Long-term mortality was not evaluated in the study (Dimick *et al*, 2003). We observed higher rates of long-term mortality at hospitals with low procedure volume in our cohort (Table 2). Volume–outcome studies are extensively used to assess variation in clinical outcomes of surgical procedures (Begg *et al*, 1998; Schrag *et al*, 2000; Birkmeyer *et al*, 2002). Hospital procedure volume has been suggested to represent part of institutional proficiency (Hodgson *et al*, 2001). The differences in outcomes could be explained in part by the variation of skills of surgeons in these hospitals. However, we observed no relation between individual surgeon case volume and overall survival (data not shown). Survival differences among hospitals with different procedure volumes may facilitate some strategies to improve quality of care. Once the top-performing centres are identified, closer examinations of detailed intraoperative or postoperative care may highlight some important factors that are relevant to prognosis and survival after hepatectomy.

We performed a population-based study using data from a large cohort that covered CLM cases from the SEER regions around the country. Most of the medical literature regarding hepatectomy for CLM consists of small single-institution, retrospective series. While powerful for examining cancer treatment on a population level, SEER-Medicare analyses rely on the use of administrative data collected by the Center for Medicare & Medicaid Services to interpret provider practices and care for individual patients tracked in the SEER tumor registry. It should also be appreciated that our study spans a relatively lengthy period of time over which surgical techniques and chemotherapy practices will have varied somewhat.

A number of important sociodemographic and other variables, such as performance status and patient preference towards treatment, are not measured by these data and thus cannot be included in predictive models to better assess important issues related to cancer outcomes, including medical decision making and patient preferences for care. Performance status has been shown to be associated with clinical outcomes, and while we controlled for comorbidities and age, poor performance status may have been associated with both access to care and poor outcome. We also do not have information on the individual serum carcinoembryonic antigen levels or the specific nature of the metastases in each patient, for example, the number of metastases, sizes of metastases, and other sites of metastases (Minagawa *et al*, 2006).

Our study confirms that the survival benefits observed in small single institutional studies of hepatic resection for CLM can be observed now on a population scale. Furthermore, hepatic resection for liver metastases can be used in elderly patients with good survival outcomes. In terms of clinical practice, our results suggest that elderly patients with resectable colorectal liver metastases who undergo surgical resection may benefit from adjuvant 5FU-based chemotherapy. It appears that both systemic chemotherapy and HAI improve outcomes after resection. In an elderly population, the outcomes of hepatic resection are impacted by hospital procedure volume, and the use of 5FU-based systemic chemotherapy and/or HAI with FUdR. The benefits of systemic therapy were recently confirmed by a randomised trial (Nordlinger *et al*, 2007). Future studies need to better delineate patient, clinician, and hospital characteristics that will lead to improved outcomes for colorectal cancer patients with surgically resectable liver metastases.

## Figures and Tables

**Table 1:** Demographic, clinical, and treatment characteristics of synchronous and metachronous patients with colorectal cancer liver metastases who underwent surgical resection, SEER-Medicare, 1991–2003

**Characteristic**	**Metachronous CLM**	**Synchronous CLM**	**All CLM**
Total	514	409	923
			
*Demographic characteristics*			
Age (years)			
65–69	178 (35%)	122 (30%)	300 (32%)
70–74	193 (38%)	134 (33%)	327 (35%)
75–79	106 (21%)	81 (20%)	187 (20%)
80+	37 (7%)	72 (18%)	109 (12%)
Race			
White	441 (86%)	361 (88%)	802 (87%)
Black	25 (5%)	26 (6%)	51 (6%)
Other	48 (9%)	22 (5%)	70 (8%)
Sex			
Female	291 (56%)	209 (51%)	500 (54%)
Male	223 (44%)	200 (49%)	423 (46%)
Socioeconomic status			
1st quintile	99 (19%)	71 (17%)	170 (18%)
2nd quintile	96 (18%)	74 (18%)	170 (18%)
3rd quintile	108 (21%)	83 (20%)	191 (21%)
4th quintile	107 (21%)	96 (23%)	203 (22%)
5th quintile	104 (19%)	85 (21%)	189 (21%)
Marital status			
Unmarried	136 (26%)	139 (34%)	275 (30%)
Married	362 (70%)	262 (64%)	624 (68%)
Unknown	16 (3%)	8 (2%)	24 (3%)
Area of residence			
Metropolitan	467 (91%)	376 (92%)	843 (91%)
Rural	47 (9%)	33 (8%)	80 (9%)
Year of diagnosis of CLM			
1991–1993	40 (8%)	64 (16%)	104 (11%)
1994–1996	96 (19%)	84 (21%)	180 (20%)
1997–1999	111 (21%)	89 (22%)	200 (22%)
2000–2003	267 (51%)	172 (42%)	439 (47%)
			
*Clinical characteristics*			
Site of primary tumour			
Rectal	146 (28%)	103 (25%)	249 (27%)
Colon	368 (72%)	306 (75%)	674 (73%)
Stage of primary colorectal cancer			
I	71 (14%)		71 (7%)
II	162 (32%)		162 (18%)
III	281 (55%)		281 30%)
IV		409 (100%)	409 (44%)
Number of positive lymph nodes			
0	210 (41%)	117 (27%)	327 (35%)
1–3	175 (34%)	153 (37%)	328 (36%)
⩾4	95 (19%)	96 (23%)	191 (21%)
Unknown	34 (7%)	43 (11%)	77 (8%)
Tumour grade of primary colorectal cancer			
Well/moderately differentiated	406 (79%)	311 (76%)	717 (78%)
Poorly/undifferentiated	90 (18%)	85 (21%)	175 (19%)
Unknown	18 (3%)	13 (3%)	31 (3%)
Comorbidity score			
0	315 (61%)	272 (67%)	587 (64%)
1	143 (28%)	97 (24%)	240 (26%)
>1	56 (11%)	40 (9%)	96 (10%)
			
*Treatment characteristics*			
Care in a teaching hospital			
No	34 (7%)	37 (9%)	71 (8%)
Yes	476 (93%)	365 (89%)	841 (91%)
Unknown	<5 (1%)	<10 (2%)	11 (1%)
Care in NCI-designated cancer centre			
No	329 (64%)	283 (76%)	612 (66%)
Yes	185 (36%)	126 (31%)	311 (34%)
Hospital procedure volume			
Low (1–8)	125 (24%)	164 (40%)	289 (31%)
Medium (9–29)	147 (29%)	120 (29%)	267 (30%)
High (>29)	242 (47%)	125 (31%)	367 (40%)
Adjuvant chemotherapy for primary cancer			
Yes	182 (35%)	NA	NA
No	332 (65%)		
Adjuvant chemotherapy after resection			
Combined FUdR and 5FU therapy	40 (8%)	34 (8%)	74 (8%)
5FU-based therapy only	187 (36%)	256 (62%)	443 (48%)
No chemotherapy	287 (56%)	122 (30%)	409 (44%)

CLM=colorectal cancer liver metastasis; 5FU=5-fluorouracil; FUdR=floxuridine; NA=not applicable.

**Table 2:** Cox proportional hazards rate ratios for overall mortality associated with demographic, clinical, and treatment characteristics

	**Metachronous CLM (*N*=514)**		**Synchronous CLM** **(*N*=409)**		**All CLMs (*N*=923)**	
**Characteristics**	**HR (95% CI)^a^**	***P*-value**	**HR (95% CI)^a^**	***P*-value**	**HR (95% CI)^a^**	***P*-value**
*Demographic characteristics*						
Age						
65–69	1.00		1.00		1.00	
70–74	1.07 (0.85–1.35)	0.65	1.14 (0.95–1.46)	0.53	**1.13 (1.00–1.28)**	**0.05**
75–79	**1.38 (1.06–1.81)**	**0.03**	1.23 (0.99–1.53)	0.13	**1.36 (1.09–1.69)**	**0.04**
80+	**1.92 (1.38–2.66)**	**<0.01**	**1.60 (1.18–2.18)**	**0.01**	**1.87 (1.46–2.34)**	**0.01**
Race						
White	1.00		1.00		1.00	
Black	1.02 (0.63–1.66)	0.44	1.07 (0.81–1.23)	0.12	1.08 (0.85–1.38)	0.24
Other	1.09 (0.83–1.46)	0.12	0.97 (0.70–1.35)	0.63	0.91 (0.74–1.12)	0.44
Sex						
Male	1.00		1.00		1.00	
Female	**0.81 (0.66–1.00)**	**0.04**	1.04 (0.88–1.24)	0.70	0.92 (0.81–1.04)	0.62
Socioeconomic status						
1st quintile	1.00		1.00		1.00	
2nd quintile	0.94 (0.67–1.31)	0.13	0.96 (0.70–1.25)	0.43	1.04 (0.86–1.26)	0.35
3rd quintile	0.92 (0.68–1.63)	0.08	0.87 (0.66–1.13)	0.37	1.14 (0.93–1.39)	0.27
4th quintile	0.83 (0.67–1.13)	0.69	0.94 (0.72–1.23)	0.11	0.92 (0.75–1.12)	0.15
5th quintile	0.87 (0.64–1.11)	0.73	0.79 (0.61–1.03)	0.34	0.96 (0.78–1.17)	0.22
Marital status						
Unmarried	1.00		1.00		1.00	
Married	0.92 (0.73–1.17)	0.86	**0.81 (0.67–0.96)**	**0.03**	0.88 (0.77–1.01)	0.05
Area of residence						
Metropolitan	1.00		1.00		1.00	
Rural	1.25 (0.84–1.89)	0.58	0.98 (0.71–1.32)	0.51	1.11 (0.88–1.38)	0.21
Year of receipt of liver resection						
1991–1993	1.00		1.00		1.00	
1994–1996	1.01 (0.59–1.50)	0.94	0.87 (0.65–1.38)	0.17	0.91 (0.76–1.08)	0.07
1997–1999	0.96 (0.69–1.80)	0.66	0.81 (0.70–1.10)	0.22	0.85 (0.71–1.03)	0.08
2000–2003	0.78 (0.60–1.12)	0.58	0.78 (0.65–1.07)	0.46	0.82 (0.67–1.05)	0.11
						
*Clinical characteristics*						
Site of primary tumour						
Rectal	1.00		1.00		1.00	
Colon	0.98 (0.61–1.57)	0.23	1.10 (0.83–1.46)	0.07	1.18 (0.98–1.28)	0.06
Tumour grade of primary cancer						
Well/moderately differentiated	1.00		1.00		1.00	
Poorly/undifferentiated	1.05 (0.78–1.41)	0.54	**1.38 (1.14–1.66)**	**<0.01**	**1.22 (1.06–1.41)**	**0.04**
Unknown	0.97 (0.53–1.76)	0.71	1.34 (0.56–2.31)	0.43	**1.32 (1.03–1.70)**	**0.03**
						
Comorbidity score						
0	1.00		1.00		1.00	
1	1.01 (0.81–1.48)	0.71	1.12 (0.87–1.43)	0.46	1.07 (0.90–1.28)	0.15
>1	1.27 (0.98–1.64)	0.39	**1.52 (1.01–2.09)**	**<0.01**	**1.29 (1.01–1.67)**	**0.05**
Number of positive lymph nodes						
0	1.00		1.00		1.00	
1–3	1.11 (0.83–1.48)	0.53	**1.81 (1.36–2.40)**	**<0.01**	**1.41 (1.18–1.68)**	**0.02**
⩾4	1.24 (0.88–1.75)	0.27	**2.21 (1.75–2.66)**	**<0.01**	**1.81 (1.51–2.19)**	**0.04**
Unknown	0.96 (0.92–1.01)	0.05	**1.06 (1.03–1.09)**	**0.04**	**1.04 (1.02–1.06)**	**<0.01**
						
*Treatment characteristics*						
Care in teaching hospital						
No	1.00		1.00		1.00	
Yes	0.97 (0.69–1.38)	0.76	0.89 (0.72–1.09)	0.12	0.93 (0.078–1.11)	0.38
Care in NCI-designated cancer centre						
No	1.00		1.00		1.00	
Yes	**0.78 (0.60–1.00)**	**0.05**	0.86 (0.67–1.07)	0.29	0.92 (0.79–1.06)	0.51
Hospital procedure volume						
Low (1–8)	1.00		1.00		1.00	
Medium (9–29)	0.83 (0.65–1.04)	0.26	**0.71 (0.55–0.92)**	**0.04**	**0.77 (0.58–0.94)**	**0.03**
High (>29)	**0.79 (0.57–0.99)**	**0.04**	**0.65 (0.50–0.88)**	**0.02**	**0.75 (0.62–0.91)**	**0.02**
Chemotherapy for primary cancer						
No	1.00		NA		NA	
Yes	**0.79 (0.62–0.97)**	**0.03**				
Chemotherapy after liver resection						
No chemotherapy	1.00		1.00		1.00	
5FU-based therapy only	0.99 (0.78–1.27)	0.10	**0.62 (0.50–0.78)**	**<0.01**	**0.76 (0.62–0.92)**	**0.02**
HAI with FUdR and systemic 5FU	0.81 (0.25–2.26)	0.92	**0.51 (0.28–0.97)**	**0.04**	0.81 (0.42–1.57)	0.52
Group						
Synchronous	N/A		N/A		1.00	
Metachronous					0.98 (0.87–1.12)	0.14

CLM=colorectal cancer liver metastasis; 5FU=5-fluorouracil; FUdR=floxuridine; HAI=hepatic arterial infusion; NA=not applicable.

a On the basis of a multiple Cox regression model in which each variable was adjusted for all others. Bold values indicate significant values.
